# Susceptibility to Declarative Memory Interference is Pronounced in Primary Insomnia

**DOI:** 10.1371/journal.pone.0057394

**Published:** 2013-02-25

**Authors:** Hermann Griessenberger, Dominik P. J. Heib, Julia Lechinger, Nikolina Luketina, Marit Petzka, Tina Moeckel, Kerstin Hoedlmoser, Manuel Schabus

**Affiliations:** Laboratory for Sleep, Cognition and Consciousness Research, Department of Psychology, University of Salzburg, Salzburg, Austria; Imperial College London, United Kingdom

## Abstract

Sleep has been shown to stabilize memory traces and to protect against competing interference in both the procedural and declarative memory domain. Here, we focused on an interference learning paradigm by testing patients with primary insomnia (N = 27) and healthy control subjects (N = 21). In two separate experimental nights with full polysomnography it was revealed that after morning interference procedural memory performance (using a finger tapping task) was not impaired in insomnia patients while declarative memory (word pair association) was decreased following interference. More specifically, we demonstrate robust associations of central sleep spindles (in N3) with motor memory susceptibility to interference as well as (cortically more widespread) fast spindle associations with declarative memory susceptibility. In general the results suggest that insufficient sleep quality does not necessarily show up in worse overnight consolidation in insomnia but may only become evident (in the declarative memory domain) when interference is imposed.

## Introduction

Positive effects of sleep on memory consolidation are compellingly demonstrated in a number of studies (for review see [1]). Repeatedly, it has been shown that memory performance is enhanced after periods of sleep relative to equal amounts of wakefulness. This evidence has been found for different kinds of memory contents. Both, procedural and declarative memories were shown to improve across periods of sleep [2]–[6] and/or to become less susceptible to interference [4], [7], [8].

As suggested by many authors in the field, the most important mechanisms behind sleep associated memory consolidation is the reactivation (and redistribution) of new memory traces during sleep (for review see [1]). Indeed, reactivation of task specific regions was found for procedural [9], [10] as well as declarative [11]–[13] memory systems. Furthermore, at least for declarative contents it has been shown that reactivations (especially in hippocampal regions) are temporally linked to thalamo-cortically generated sleep spindles [14]–[17]. The electrophysiological pattern of the sleep spindle can lead to plastic changes at the neocortical level (e.g. [18]) and thus, may serve as a marker for successful memory consolidation during sleep. This involves the redistribution of new and fragile memory traces into stable cortical memory systems which lead to facilitated long term accessibility and reduced susceptibility to interference. Although there is no evidence for a direct coupling of spindles and the reactivation of procedural memories during sleep, especially sleep spindles over sensorimotor regions have been found to be related to the consolidation success of procedural skills [19]. Note that in general spindles are more and more discussed as local, experience-dependent phenomena. For the declarative memory domain, and in accordance with known functional specializations, Clemens et al. [20] for example reported spindle foci over left frontocentral areas for verbal declarative material.

Most studies addressing the impact of sleep on memory consolidation traditionally focus on healthy sleepers. Yet it is interesting to study if and in which way disturbed sleep would change the mentioned overnight memory consolidation. Primary insomnia is a widespread health problem which affects approximately 5–10% of the adult population [21], [22]. Patients with insomnia have difficulties falling or staying asleep, or report non-restorative sleep and significant daytime impairments [23]. In addition, decreased quality of life and complaints in cognitive functioning are frequently reported by these patients [24], [25]. Backhaus and colleagues [26] tested whether people suffering from primary insomnia show greater impairments in a declarative memory task as compared to healthy control sleepers. Their main finding was that patients displayed less overnight declarative memory consolidation and that the amount of slow wave sleep was significantly correlated with efficient overnight consolidation in control subjects only. Interestingly, procedural memory consolidation as measured by a mirror tracing task did not reveal any differences between the healthy and the patient group. Contrary to these data, Nissen and colleagues [27] only found impaired procedural memory consolidation in primary insomniacs, but no significant differences in declarative memory consolidation over sleep. Interestingly, also other studies addressing cognitive performance in insomnia (for review see [28], [29]) report small to medium sized impairments despite considerable subjective complaints. The main objective of the present study was thus to investigate the relationship between sleep and memory consolidation in primary insomnia using a new, and so far in insomnia research not used approach, namely the evaluation of memory susceptibility to interference. Given the hampered sleep quality in people suffering from insomnia we hypothesized that insomnia patients would specifically display pronounced overnight forgetting following interference manipulations.

## Materials and Methods

### Ethics Statement

Data were obtained from a larger investigation comparing primary insomnia patients and healthy sleepers and the influence of instrumental sensorimotor rhythm conditioning (DRKS0003265). The study protocol was approved by the local ethics committee (“Ethikkommission Paris Lodron-Universität Salzburg”) and the Declaration of Helsinki. Participants gave written informed consent after study briefing. Data presented here were not used in other publications to date.

### Subjects

For the present study twenty-seven patients with primary insomnia (mean age = 36.67 years, SD = 10.54) and twenty one age and sex matched healthy subjects (mean age = 33.53 years; SD = 9.75) were examined (N = 48). All of the subjects were non-habitual smokers (less than 5 cigarettes a day) and were free of any medication for at least 2 weeks prior to the onset of the study. They underwent a thorough entrance examination including Diagnosis of Psychiatric Disorders according to DSM-IV (Structured Clinical Interview for DSM disorders) and psychometric tests (e.g. personality, intelligence). Primary insomnia was classified according to the research diagnostic criteria of Edinger and colleagues [30]. Actigraphy (Cambridge Neurotechnology Actiwatch ©) and sleep diaries were used to control for regular sleep wake rhythms in patients (starting at least 1 week before the learning night and ending with study completion). Healthy subjects were regular sleepers as verified by clinical interviews and the Pittsburgh Sleep Quality Index questionnaire [(PSQI-score<5)] and were included only if their minimum sleep efficiency was not more than one standard deviation below the average sleep efficiency in age and sex matched healthy sleepers (according to an European data base [31]: M = 88.97; SD = 6.7).

### Procedure

After an entrance examination subjects spent a screening/adaptation night in the laboratory to ensure that they had no other sleep disorders (sleep apnea, bruxism, periodic leg movements etc.) besides insomnia and to familiarize subjects with laboratory conditions. Thereafter, two experimental nights were recorded. Experimental nights were preceded by either learning a procedural (finger tapping task, FTT) or a declarative memory task (word pair associations, WORDS; for more details please refer to task descriptions below). The order of the experimental nights (FTT vs. WORDS) was counterbalanced between subjects.

**Figure 1:**
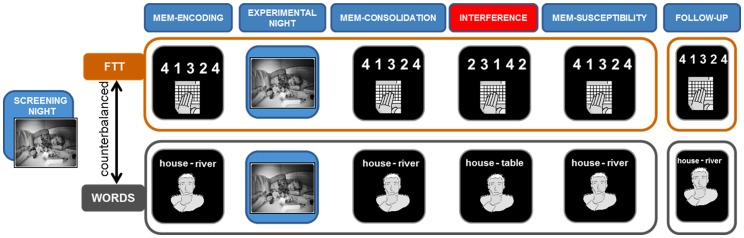
Experimental design. All subjects slept three nights in the sleep laboratory. Whereas the first night was only used for screening and adaptation purposes, nights 2 and 3 served as experimental nights (EXPERIMENTAL NIGHT). Preceding each EXPERIMENTAL NIGHT, subjects either learned (MEMORY ENCODING)the finger tapping task (FTT) or the declarative memory task (WORDS). Note that the order of the tasks were counterbalanced within subjects. In the morning (for either task) a cued recall testing for overnight memory consolidation (MEM-CONSOLIDATION) was followed by an interfering learning session (INTERFERENCE). Thereafter, participants were retested for the initial learning material from the previous day (MEM-SUCEPTIBILITY). A Follow up testing session was done in a subgroup of the study population to control for long-term effects (FOLLOW-UP).

Subject’s overnight memory change [MEM-CONSOLIDATION] was tested in the morning after eight hours of time in bed. Subsequently, all participants had to perform an interfering task [INTERFERENCE]. In the procedural task participants trained a new FTT sequence and in the WORDS condition subjects had to learn new word associations (e.g., house – TABLE instead of house – RIVER). After a 20 min break, subjects were then retested for the initially learned FTT sequences or word pair associations (house – RIVER) in order to examine subject’s interference susceptibility [MEM-SUSCEPTIBILITY]. Additionally, we tested both groups of control and insomnia subjects (Controls: N = 11 [FTT and WORDS]; Insomnia Patients: N = 17 [FTT], N = 16 [WORDS]) for long-term memory retention within 5–8 days after the second experimental night [FOLLOW-UP]. Figure 1 illustrates the study design and the various testing sessions.

Before bedtime and in the morning after learning, subjects were asked to fill out questionnaires regarding subjective sleepiness (Stanford Sleepiness Scale; SSS [32]) and mood (Mehrdimensionaler Befindlichkeitsfragebogen; MDBF [33]) and performed a simple cued reaction time task (psychomotor vigilance task; PVT [34]).

### Procedural Memory Task

To investigate procedural memory consolidation a classical motor skill paradigm, the so-called finger tapping task (FTT), was chosen. Participants were given a specific five element sequence (4 1 3 2 4) which they had to train by continuously pressing the respective numeric keys on a computer keyboard with the non-dominant hand. At first participants completed a trial run to ensure that the appropriate buttons are used. Afterwards, participants had to repeat the test sequence (4 1 3 2 4) for a duration of 30 sec as quickly and accurately as possible, followed by a 30 sec rest period. All subjects trained the test sequence 12 times in 30 sec blocks in the evening [MEM-ENCODING] to acquire the motor skill (for further information see [4]). The following morning subjects were retested on three trials on the previously trained sequence (4 1 3 2 4) to investigate the effect of sleep on overnight procedural memory performance [MEM-CONSOLIDATION]. Subsequently an interference sequence (2 3 1 4 2) was trained (analogues to above) followed by a 20 min break. Finally, participants were retested on the original FTT sequence (4 1 3 2 4) in order to evaluate susceptibility to interference [MEM-SUSCEPTIBILITY].

Behavioural measures capture speed and accuracy and are calculated as the number of correctly generated three-element chunks during the 30 sec period of a block [35]. The task was implemented in MATLAB (The MathWork 7.14 R2012a) and presented on a 15.6″ monitor.

### Declarative Memory Task

Declarative memory was investigated using a word-pair association task with pairs of nouns being presented visually on a 15.6″ monitor. The combination of word-pairs was randomized for each participant. Analogous to the FTT procedure participants studied 60 word-pairs (list A–B) in the evening before the experimental night [MEM-ENCODING]. Each word-pair was presented once for 7 sec. Immediately after learning, all subjects performed a cued-recall of the previously learned word-pairs with the first word of each pair (A-word) serving as cue. Participants should then report the corresponding second word (B-word) within 10 sec. The participants received immediate feedback by appearance of the correct word if they did not recall the corresponding word (for further detail see [8]). Subjects studied the material until at least 70% of words were recalled correctly. Regardless of the 70% threshold all 60 A-words were presented at least once. If participants did not reach the criterion in the first run, all incorrectly retrieved words were presented again until at least 70% of the 60 words were recalled correctly.

The next morning declarative memory consolidation was tested [MEM-CONSOLIDATION] with 20 randomly selected word-pairs of the original A–B word-list. Afterwards, the interference condition started. Participants studied 40 new word-pairs with the first words (A-words) being identical (e.g., original: A–B [house – RIVER]; interference: A–C [house – TABLE]). Training was analogous to the evening session. Finally after a 20 min break subjects were asked to recall both B and C words with the A-words of the last 40 word-pairs again serving as cue [MEM-SUSCEPTIBILITY]. In addition, a follow up test was introduced for a subgroup of control and insomnia participants in order to test for long-term memory retention (A–B and A–C pairings) [FOLLOW-UP].

### EEG Recordings

Synamps EEG amplifiers (NeuroScan Inc., El Paso, Texas) were utilized to record the electroencephalogram (EEG). The signals were filtered with a 0.10 Hz high-pass filter, a 70 Hz low-pass filter and a 50 Hz notch filter to avoid interfering signals. The sampling rate for online digitization was set to 500 Hz. For task EEG recordings 23 scalp EEG channels (Fp1, Fpz, Fp2, F3, Fz, F4, F8, T3, C3, Cz, C4, T4, T5, P3, Pz, P4, T6, O1, Oz, O2 plus A1 and A2 for later re-referencing), 1 bipolar vertical (VEOG) and 1 bipolar horizontal electrooculogram (HEOG) for later eye artifact corrections, 1 electromyogram (EMG) channel, 1 electrocardiogram (ECG) channel and 1 respiratory channel (chest wall movements) were placed. Scalp electrodes were adjusted according to the international 10–20 system [36]. Polysomnography (PSG) during screening nights included 8 EEG, 4 EOG, 1 bipolar ECG, 4 unipolar EMG (submental and left/right tibialis) and 4 respiratory channels (nasal airflow, chest and abdominal wall movements, oxygen saturation). Sleep was scored automatically by the SOMNOLYZER 24*7 (The Siesta Group ©) and verified manually by a sleep scoring expert following sleep scoring criteria of the American Academy of Sleep Medicine (AASM).

### Sleep Spindles in N2 and N3

Sleep spindles were detected automatically at two frontal (F3, F4), two central (C3, C4) as well as two parietal (P3, P4) electrodes. For spindle detection the following criteria were used: (1) 11–15 Hz band pass filtering, (2) amplitude >25 µV, duration >0.5 secs and (4) correction for muscle (30–40 Hz) and/or alpha artefacts (8–12 Hz). For further details see information from Anderer and colleagues [31]. Moreover, we divided spindles into a slow range (11–13 Hz) and fast range (13–15 Hz) [37]. The utilized spindle activity measure (spindle activity; SpA [38]) is based on the duration and amplitude of all identified spindles during sleep stages 2 (N2) and slow wave sleep (N3; separately calculated). Thus, this measurement reflects the activity or intensity of the spindle process (spindle activity = mean spindle duration * mean spindle amplitude). We also added a difference score (C4–C3) for spindles in order to control for possible lateralization effects after non-dominant (left) hand FTT completion (cf. Table S1).

### Statistics

Statistical analyses were performed using SPSS 18 software (SPSS Inc., Chicago, Illinois). Descriptive data of objective and subjective sleep parameters are presented by mean values and standard deviations. T-tests were used to assess behavioural differences between healthy sleepers and people suffering from primary insomnia.

For calculations of the finger tapping task we built the mean values of the last three training blocks in the evening, the morning recall, the morning recall after interference and the interference paradigm (for block-by-block FTT results please see Figure S1). Performance differences in the finger tapping task were analysed with 2×2 ANOVAs. One with the focus on overnight change included factor GROUP (primary insomnia vs. healthy controls) and factor TIME1 (MEM-ENCODING vs. MEM-CONSOLIDATION) the other with the main focus on the interference paradigm included factor GROUP (primary insomnia, healthy controls) and factor TIME2 (MEM-CONSOLIDATION vs. MEM-SUSCEPTIBILITY). For the follow up testing subgroup we calculated a 2×2 ANOVA with factor GROUP (primary insomnia, healthy controls) and factor TIME3 (MEM-SUSCEPTIBILITY vs. FOLLOW UP). Additionally, t-tests were calculated post hoc in order to quantify the degree of interference learning between groups.

Pearson correlation-coefficients were used to explore the relationships between sleep parameters (sleep efficiency, sleep onset latency, time awake after sleep onset, number of awakenings, stage N1, stage N2, stage N3 and rapid eye movement sleep [R]), sleep spindles and overnight memory changes. Overnight memory change 1 (OMC 1) was defined as difference scores between initial learning in the evening (MEM-ENCODING) and morning retrieval after awakening, before interference (MEM-CONSOLIDATION). Overnight memory change 2 (OMC 2) was defined as difference score from initial learning (MEM-ENCODING) to retrieval after interference manipulation (MEM-SUSCEPTIBILITY).

Declarative memory scores were defined as percentage of correct answers (60 MEM-ENCODING word-pairs, 20 MEM-CONSOLIDATION word pairs and 40 MEM-SUSCEPTIBILITY word pairs). ANOVAs, post hoc tests and correlations of the same type and following an identical logic were calculated for the declarative memory domain (for details on A–C interference retrieval scores refer to Figure S2). For the declarative memory task we had to exclude two insomnia patients and one healthy control subject due to missing data in the evening. The level of significance was set to p<0.05 (two-tailed).

## Results

### Sleep EEG

Sleep data from the experimental nights (FTT, WORDS) are shown in Table 1. Analysis for the FTT night revealed significant differences between insomnia patients and controls in sleep efficiency (t_46_ = 2.28, p = 0.028), total sleep time (t_46_ = 2.25, p = 0.031), and wake after sleep onset (WASO) (t_46_ = −2.61, p = 0.014) indicating better sleep quality in healthy sleepers (see Table 1). Regarding the experimental night following declarative word-pair learning differences in sleep parameters between healthy controls and insomnia patients were found for sleep efficiency (t_43_ = 2.818, p = 0.008) and WASO (t_43_ = −3.259, p = 0.003) again indicating better sleep quality for healthy sleepers. Differences in total sleep time just failed to reach significance (t_43_ = 1.846, p = 0.072).

**Table 1:** Sleep parameters for healthy controls and primary insomnia patients separated for both experimental nights (finger tapping task, FTT; declarative word pair task, WORDS).

FTT	Controls (n = 21)			Patients(n = 27)			t	p
Time in Bed (min)	480.14	±	4.71	478.06	±	27.35	0.35	0.732
Total sleep time (min)	439.57	±	23.18	409.94	±	63.11	2.25	0.031*
Sleep efficiency %	91.56	±	5.00	85.73	±	11.97	2.29	0.028*
Sleep onset latency (min)	17.93	±	16.55	18.09	±	23.39	−0.03	0.978
R Latency (min)	88.33	±	36.97	110.85	±	60.26	−1.59	0.118
Wake Time (min)	22.26	±	13.41	49.80	±	52.58	−2.61	0.014*
Number of awakenings	18.95	±	8.31	21.19	±	12.05	−0.72	0.472
N1%	13.11	±	6.46	13.22	±	5.76	−0.06	0.951
N2%	46.99	±	7.72	45.40	±	8.51	0.67	0.506
N3%	18.80	±	8.33	21.34	±	7.31	−1.12	0.268
R%	21.10	±	5.10	20.04	±	6.77	0.59	0.556
**WORDS**	**Controls** **(n = 20)**			**Patients** **(n = 25)**			**t**	**p**
Time in Bed (min)	470.20	±	29.97	477.58	±	16.63	−1.05	0.301
Total sleep time (min)	430.30	±	36.28	405.38	±	50.85	1.85	0.072
Sleep efficiency %	91.54	±	4.75	84.91	±	10.49	2.82	0.008**
Sleep onset latency (min)	14.50	±	12.76	14.34	±	12.36	0.04	0.966
R Latency (min)	90.23	±	44.10	95.64	±	50.59	−0.38	0.708
Wake Time (min)	25.10	±	18.35	57.12	±	44.66	−3.26	0.003**
Number of awakenings	17.00	±	8.71	20.96	±	8.13	−1.57	0.123
N1%	13.39	±	6.57	13.40	±	5.47	−0.01	0.994
N2%	48.22	±	9.61	45.31	±	5.64	1.20	0.239
N3%	18.28	±	10.18	21.45	±	6.87	−1.24	0.220
R%	20.11	±	5.46	19.84	±	5.73	0.16	0.876

Values are means ± standard deviations (SD). R, rapid eye movement; N1, stage N1; N2, stage N2; N3, stage N3;

* p<0.05,

** p<0.01.

Directly comparing the experimental nights after FTT and WORDS revealed no significant differences in sleep parameters (time in bed, total sleep time, number of awakenings, sleep efficiency, sleep stages) for the control group and insomnia patients.

### Finger Tapping Task (FTT)

**Figure 2:**
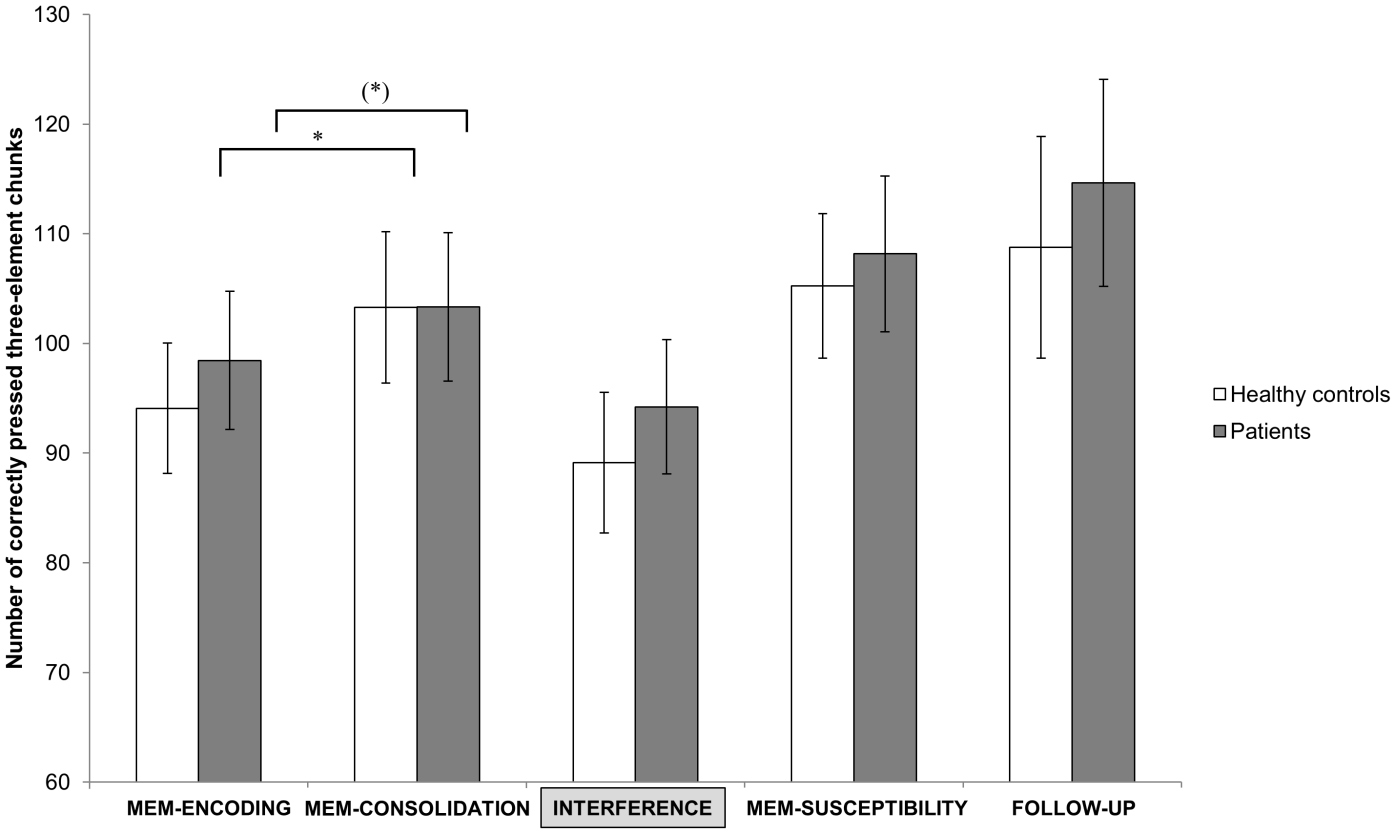
Finger-tapping results. Bars represent the mean number of correct three element chunks (± standard errors) during MEM-ENCODING, pre-interference (MEM-CONSOLIDATION), at INTERFERENCE, at post-interference (MEM-SUSCEPTIBILITY) and at FOLLOW-UP. Significant results are indicated by asterisks. *p<0.01; (*), p<0.1. Note that a subgroup (N = 28) was also tested for long-term retention (FOLLOW-UP) and that there are no performance differences between insomnia patients and the control group.

Overall, behavioral effects revealed a main effect for overnight performance change (TIME 1) (F(1,46) = 15.198, p<0.001) indicating an increase in performance in healthy controls (t_20_ = −3.86, p = 0.001; mean evening = 94.08±27.26, mean morning = 103.27±31.63) and a trend toward an increase in insomnia patients (t_26_ = −1.88, p = 0.070; mean evening = 98.44±32.78; mean morning = 103.33±35.24; cf. Figure 2) but no significant interaction (GROUP x TIME1). There was no difference in interference learning between insomnia and control subjects (t_46_ = −5.73, p = 0.573).

No systematic change in performance from pre- to post interference (TIME2) (F(1,46) = 1.979, p = 0.166) nor a significant interaction (GROUP * TIME 2) (F(1,46) = 0.345, p = 0.560) (cf. Figure 2) could be found. Follow up testing revealed neither a significant main effect (TIME 3) (F(1,26) = 0.873, p = 0.359) nor an interaction (GROUP × TIME 3) (F(1,26) = 0.014, p = 0.907).

We did not find any significant relationships between overnight memory change 1 (MEM-CONSOLIDATION – MEM-ENCODING) and overnight memory change 2 (MEM-SUSCEPTIBILITY – MEM-ENCODING) with sleep parameters in the insomnia group. In the control group relationships between overnight memory change 1 and N1% (r = .451, p = 0.04) and between overnight memory change 2 and REM % (r = –.477, p = 0.029) were revealed (see Table 2).

**Table 2:** Correlation coefficients between memory change scores and sleep parameters.

		SEFF %	SOL(min)	WASO (min)	NOA	N2%	N3%	R%	N2 SpAslow	N2 SpAfast	N3 SpAslow	N3 SpAfast
**FTT**												
controls	OMC 1	−0.177	0.400(*)	−0.159	0.265	−0.047	−0.177	−0.211	0.320	0.317	0.148	0.293
	OMC 2	0.207	0.001	−0.353	−0.136	0.038	0.049	−0.477*	0.221	0.217	0.508*	0.459*
patients	OMC 1	0.000	−0.014	0.022	0.018	−0.223	0.249	−0.042	0.090	0.077	0.064	0.128
	OMC 2	0.194	−0.228	−0.094	0.135	−0.191	0.168	−0.129	−0.027	0.143	−0.101	0.119
**WORDS**												
controls	OMC 1	0.432	−0.270	−0.263	−0.328	−0.111	−0.109	0.511*	−0.300	−0.236	−0.102	−0.086
	OMC 2	0.270	0.200	−0.462*	−0.328	0.259	−0.068	0.147	0.162	0.062	0.315	0.292
patients	OMC 1	0.292	−0.157	−0.276	−0.209	−0.164	−0.072	−0.073	−0.162	−0.192	0.059	−0.052
	OMC 2	0.235	0.160	−0.278	−0.561**	0.044	0.097	−0.195	0.317	0.340	0.431*	0.417*

OMC1 = overnight memory change 1 (MEM-CONSOLIDATION – MEM-ENCODING); OMC2 = overnight memory change 2 (MEM-SUSCEPTIBILITY – MEM-ENCODING); SEFF = sleep efficiency; SOL = sleep onset latency; WASO = wake after sleep onset; NOA = number of awakenings; N2 = stage 2 sleep; N3 = slow-wave sleep; R = rapid eye movement sleep; SpA = spindle activity. (*)p<0.1, *p<0.05, **p<0.01. Note that only representative electrodes are provided for SpA (FTT: C4, WORD: F3), for additional details please refer to the supplementary material.

**Figure 3:**
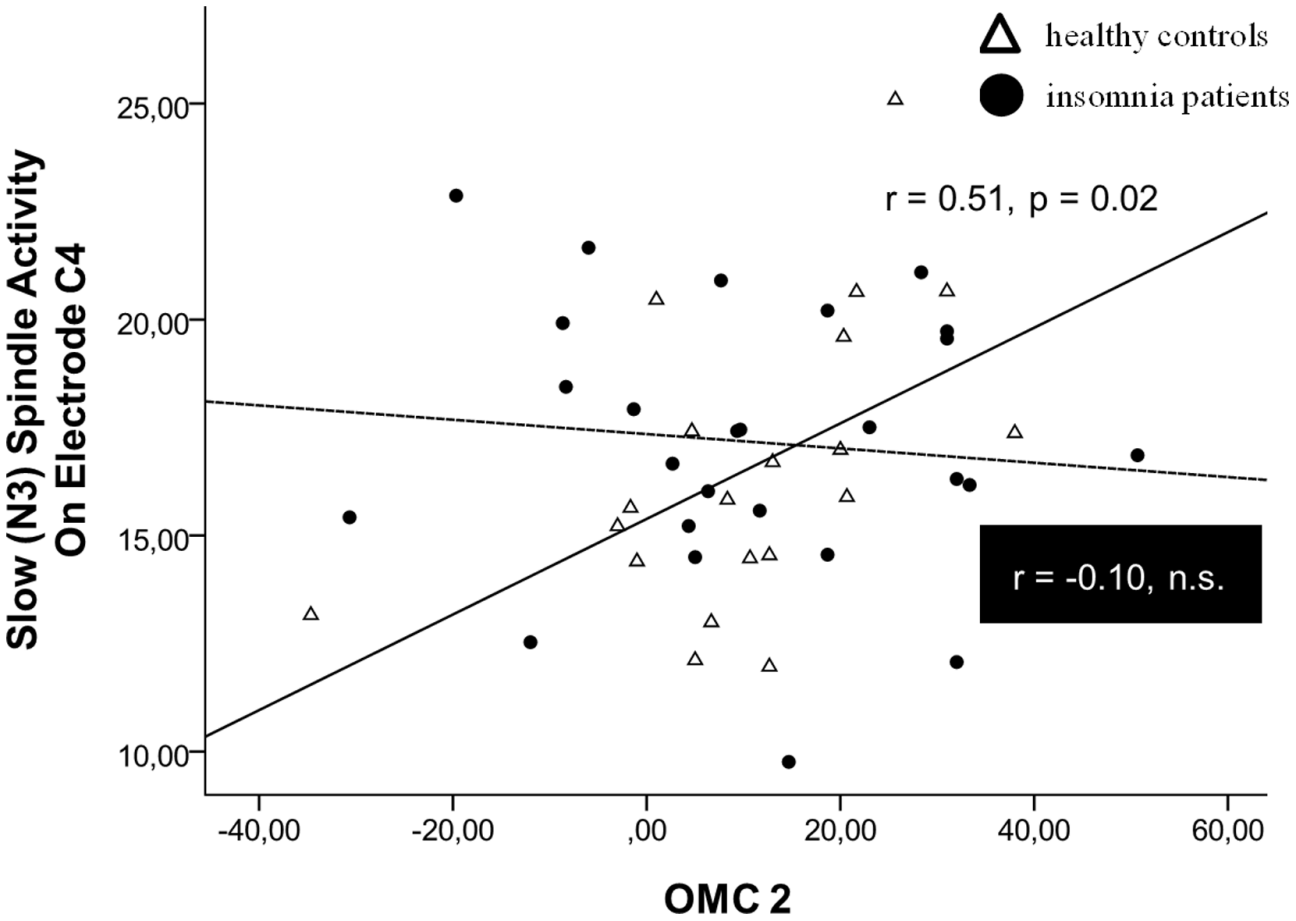
Overnight memory change (procedural learning) and sleep spindles. Correlation between procedural (FTT) performance change across interference (OMC 2 = overnight memory change 2; MEM-SUSCEPTIBILITY – MEM_ENCODING) and slow spindle activity (over central electrode C4) in N3 sleep separated for controls (solid regression line) and insomnia patients (dashed regression line). Note that all subjects were performing the FTT using the non-dominant (left) hand.

With regard to correlations between overnight memory change 1 & 2 and sleep spindles significant relationships were only revealed for control subjects. Controls showed a significant positive correlation between slow spindle activity in N3 and overnight memory change 2 (C3: r = .545, p = 0.011; C4: r = .508, p = 0.022, cf. Figure 3). Furthermore, fast spindle activity in N3 was related to overnight memory change 2 (C4: r = 0.459, p = 0.037). Insomnia patients did not show any such associations with sleep spindles (for details also refer to Table S2).

### Declarative Word Pair Task

**Figure 4:**
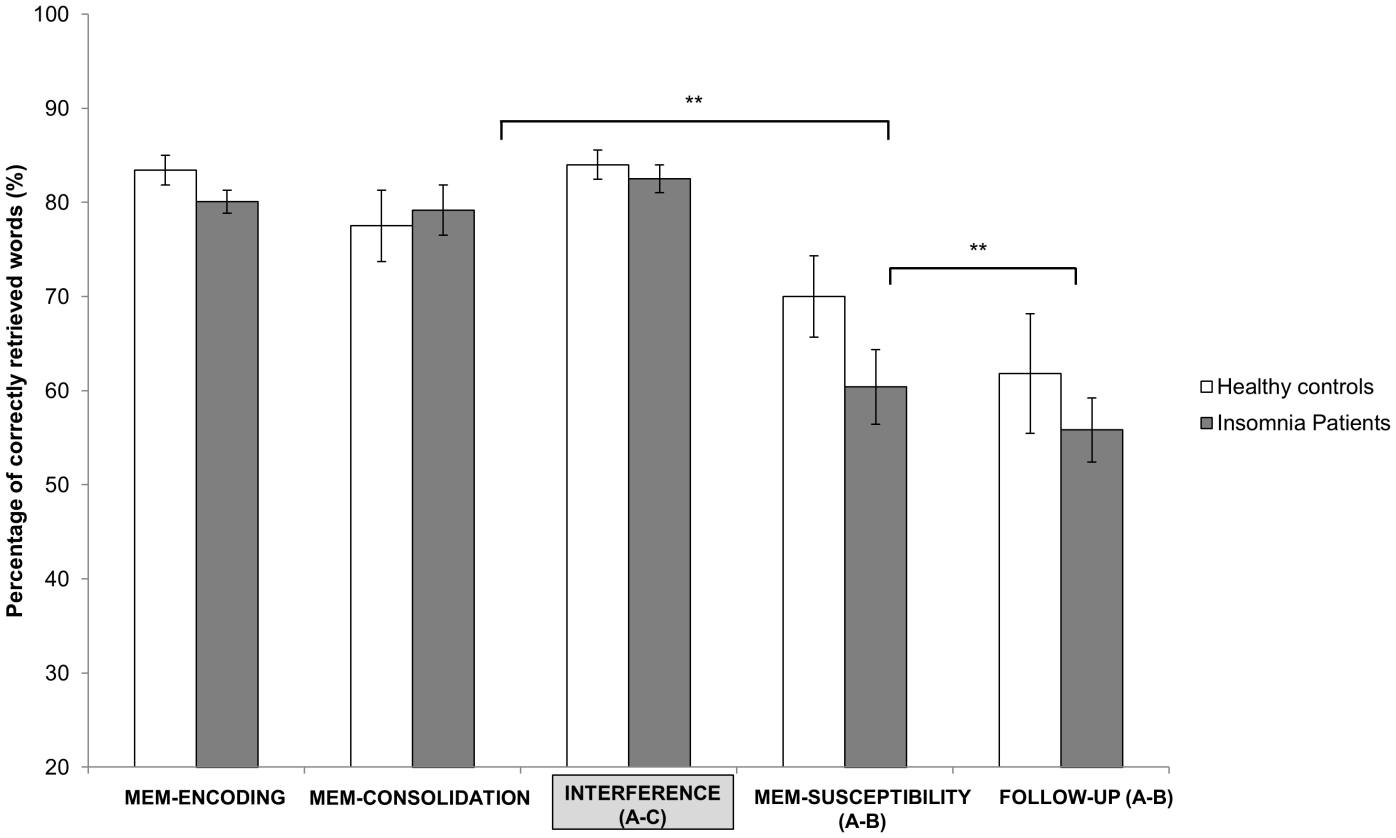
Word-pair-task results. Declarative verbal memory scores from MEM-ENCODING, subsequent morning recall (MEM-CONSOLIDATION), interference learning (INTERFERENCE), morning recall after interference (MEM-SUSCEPTIBILITY) and follow up (FOLLOW-UP; only a subgroup was tested). Bars represent means ± standard errors. Significant results are indicated by asterisks. **p<0.01. Note that only insomnia patients show significant forgetting after interference learning.

Regarding overnight memory change no significant effects could be revealed for factor TIME 1 (F(2,43) = 2.51, p = 0.12) nor for the interaction (GROUP × TIME 1) (F(1,43 = 1.391, p = 0.245). The ANOVA addressing the interference effects revealed a main effect for factor TIME 2 (F(1,43) = 28.182, p<0.001) as well as a significant GROUP × TIME 2 interaction (F(1,43) = 5.203, p = 0.028). These results indicate that while both groups showed less memory retention after the interference task, the magnitude of forgetting was greater in the insomnia group (t_24_ = 5.7, p<0.001; MEM-CONSOLIDATION % = 79.2±13.36; MEM-SUSCEPTIBILITY % = 60.4±19.83) than in the healthy group (t_19_ = 2.027, p = 0.057; MEM-CONSOLIDATION % = :77.50±16.81; MEM-SUSCEPTIBILITY% = 70.00±19.26) (see Figure 4). Interference learning was identical for insomnia and control subjects (t_43_ = 6.90, p = 0.494). Follow up testing revealed further forgetting from MEM-SUSCEPTIBLITY to FOLLOW-UP [TIME 3] TIME 3 (F(1,25) = 15.217, p = 0.001) with the effect being mainly mediated by the insomnia group (insomnia group: t_15_ = 4.616, p<0.001; control group: t_10_ = 1.691, p = 0.122).

**Figure 5:**
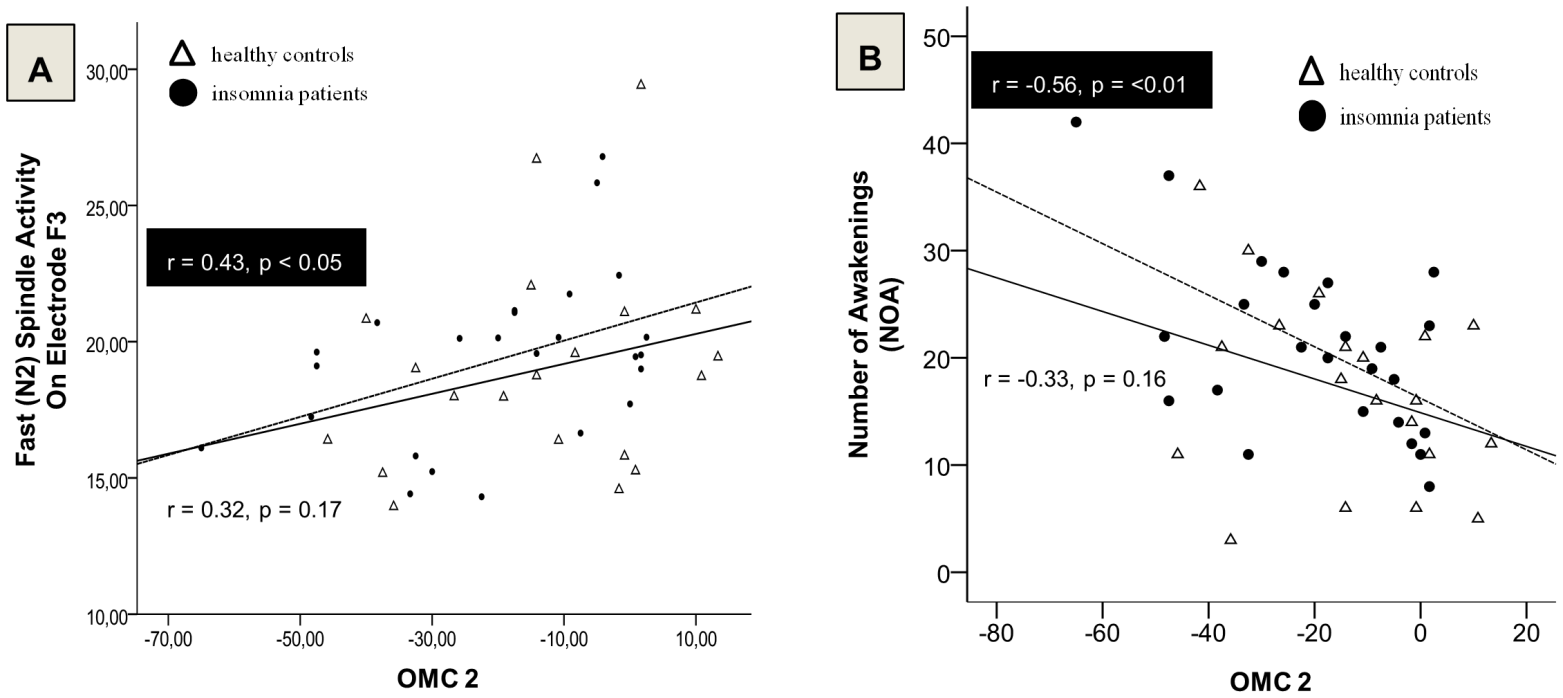
Declarative overnight memory change after interference. (A) Fast spindle activity (N2 sleep) at frontal recording site F3 is positively related to overnight memory change 2, that is from initial learning (MEM-ENCODING) to post-interference testing (MEM-SUSCEPTIBILITY) in insomnia patients (dashed line). *(B)* Relationship between overnight memory change 2 and number of awakenings. Note that frequent awakening is related to more pronounced forgetting after interference learning in insomnia patients (dashed regression line).

In addition, analysis revealed a relationship between overnight memory change 1 and REM sleep (r = .511, p = 0.021) as well as a negative correlation between overnight memory change 2 and WASO (r = –.462, p = 0.04) in the control group. In the insomnia group overnight memory change 2 was negatively associated with the number of awakenings (r = –.561, p = 0.004, see Figure 5). These results suggest that higher sleep fragmentation characterized by a greater number or longer duration of nightly awakenings is associated with decreased memory stability and higher susceptibility to interference.

Correlation analysis revealed associations of mainly fast sleep spindles and overnight memory change 2 for insomnia patients (F3_N2-sleep_: r = .431, p = 0.032, see Figure 5; F3_N3-sleep_: r = .417, p = 0.038; F4_N2-sleep_: r = .410, p = 0.042; F4_N3-sleep_: r = .398, p = 0.049; C4_N3-sleep_: r = .413, p = 0.040), as well as one significant correlation with slow spindles (P4_N3-sleep_: r = .467, p = 0.028). For controls only one trend for fast spindles was found (C3_N2-sleep_: r = .390, p = 0.085) (for full details please refer to Table S2).

## Discussion

This study investigated the relationships between sleep, memory consolidation and susceptibility to interference in patients suffering from primary insomnia as well as in healthy sleepers. In the procedural memory domain (FTT) the healthy control group showed a significant overnight enhancement. Here, even insomnia patients exhibited a trend in the same direction although sleep quality was significantly deteriorated. In the procedural memory task an interference manipulation the next morning did not affect any of the groups. Interestingly, in the declarative memory domain a completely different picture emerged. Although overnight memory consolidation was not different between groups, insomnia patients demonstrated significant memory deterioration (A–B word pairings) after an intervening interference session (A–C pairings).

It is interesting to note that in our study even an interfering tapping sequence applied during the next morning did not diminish the previously learned procedural memory sequence. It may appear counterintuitive that insomnia patients, who show chronically less sleep efficiency, less total sleep time and more time awake as compared to healthy controls (cf. Table 1), exhibit nearly identical amounts of overnight gains in motor skills and specifically no susceptibility to interference. Therefore, it could be concluded that the residual amounts of sleep in patients suffering from insomnia may be sufficient for successful motor memory consolidation or that sleep is not needed at all in order to consolidate this specific procedural skill [5], [39], [40]. The high performance after awakening also fits well with the idea of hyperarousal as a compensatory mechanism in insomnia patients [41]–[43]. According to this theory cognitive hyperarousal helps insomnia patients to compensate self-reported daytime complaints and cognitive deficits and, therefore, often masks performance decrements.

Note that while some studies report procedural overnight memory decrements in insomnia [27], other studies using the FTT also did not find much affected procedural skills [44]. In accordance with our findings a recent meta-analysis [28] concluded that motor skill memory is indeed most often objectively not found to be impaired in insomnia patients. With regard to our healthy controls, results are consistent with other findings describing that sleep in healthy sleepers can additionally boost procedural memory consolidation [45]–[47]. While some studies suggest that overnight performance gains are correlated with the amount of non-REM (e.g. [48]) or REM sleep [3], [49], we did not find such correlations. Even more surprisingly we found negative associations of REM sleep and memory change across interference (overnight memory change 2), indicating that post-learning REM makes the initial memory trace more susceptible to interference.

Concerning more fine-grained sleep mechanisms we analyzed N2 and N3 sleep spindles separately for both groups. Although we did not observe relationships between spindle activity and procedural performance gains in the insomnia group, healthy controls exhibited an association between (slow and fast) spindle activity in deep slow-wave sleep and memory change across interference (OMC 2). The importance of sleep spindles on motor tasks has been reported repeatedly (e.g. [50]–[53]) with fast spindles appearing to play a specific role. At first sight some of our findings seem to contradict these earlier reports. However given a recent publication by Mölle and colleagues [54] it appears possible that also the revealed “slow” spindle effects in N3 sleep might be in accordance with earlier “fast” spindle findings (in N2) as Mölle and colleagues demonstrate that spindles tend to decrease in frequency in deeper sleep stages. It is also interesting to note that our found spindle relationships for procedural memory are localized over sensorimotor regions (C3, C4) in agreement with the known functional specialisation. Together with the negative association of REM sleep and overnight memory performance change our findings favor the view that REM sleep is not necessarily critical for the consolidation of this specific procedural motor skill [55]–[57] but perhaps rather sleep mechanisms such as N3 sleep spindles. Which alternative sleep mechanism might be utilized for procedural consolidation in insomnia patients has to be revealed. In the present sample we could not identify any such mechanisms.

Interestingly, in the declarative memory task the interference effect was revealed as hypothesized. Although insomnia patients did not generally show more overnight forgetting than healthy controls it could be demonstrated that interference (A–C) learning on the next morning deteriorates their performance levels more significantly. That is, insomnia patients are worse in recalling initially encoded memories after interference was imposed in the morning hours. Furthermore delayed recall (after 5–8 days) verified this more pronounced forgetting in insomnia patients.

The susceptibility of memory to interference in patients suffering from primary insomnia is well in line with recent work indicating that disturbed sleep is associated with a diminished sleep related declarative memory consolidation [26].

The question remains if any specific sleep parameter may predict the susceptibility of declarative memories to next day interference. For this purpose we looked at classical sleep parameters such as sleep stages as well as sleep spindle activity.

Regarding spindle activity we found consistent relations of overnight memory change 2 and fast sleep spindles over left frontal brain regions (F3) in agreement with earlier reports using verbal declarative material [20]. However, further non-location specific associations (over F4, C4, P4) indicate a cortically more widespread consolidation process for declarative memories, which also may built upon light NREM as well as deep sleep consolidation mechanisms. To date it is unclear why these effects were only revealed in our insomnia sample. We believe that simply statistical power may be one reason as fast frontal spindles show associations of the same direction in healthy individuals.

Regarding the measured sleep parameters following the declarative memory task it was revealed that especially WASO time and number of awakenings were strongly correlated with susceptibility to interference. This might be of considerable relevance as it indicates that non-restorative sleep, in our case “only” an increase of 30 min wake time during sleep, may already have significant impact on the stability of previously learned material. This further emphasizes the importance of attaining undisturbed sleep in well controlled sleeping environments also in healthy individuals. Furthermore, it has to be noted that in healthy controls a positive relationship between overnight memory change and REM sleep was found. As only few studies have shown a dependence of declarative memory consolidation on REM sleep (for reviews see [58]–[60]) our results add a further fragment to a yet incomplete picture of sleep-related memory consolidation [61].

One limitation of the study is the surprisingly moderate sleep quality of our healthy individuals. This is specifically expressed by an average sleep efficiency of 91.55% and the moderate sleep onset latency (16.22 min) presumably related to the higher age range (20 to 57) of the present study sample. Furthermore, it has to be noted that due to our strict study criteria of primary insomnia [30] we presumably were biased in selecting patients with sleep continuity problems rather than sleep onset problems.

### Conclusion

In summary, the results suggest that memory consolidation in insomnia patients is not affected in the procedural (i.e. finger tapping) but rather the declarative memory domain (word-pair learning) if memory is tested after a morning interference manipulation. Focusing on the role of sleep, our findings demonstrate that bad sleep quality, as evidenced by prolonged WASO time or enhanced number of awakenings, is negatively affecting overnight memory stability.

With regards to sleep spindles the general picture emerging is a locally specific (sensorimotor) consolidation of motor memories in slow-wave sleep, as well as a cortically more widespread declarative memory consolidation during fast spindles in light as well as deep sleep. Successful treatments to improve sleep quality and maybe even sleep spindles directly may, thus help to make new experiences less susceptible to interference in all sleepers.

## Supporting Information

Figure S1 **Performance during training (blocks 1–12), subsequent morning retest (blocks 13–15; in a box), interference testing (blocks 16–27), morning retest after interference (blocks 28–30; in a box) and follow up testing (blocks 31–33).** Note that only a subgroup was tested in the follow up.
(TIF)

Figure S2 **Declarative verbal memory scores from the evening, subsequent morning recall (MORNING 1), interference learning (INT), morning recall after interference (MORNING 2) and follow up (only a subgroup was tested) for all word lists (A–B, A–C).** Bars represent means ± standard errors. Significant results are indicated by asterisks. **p<0.01. Note that only insomnia patients show significant forgetting after interference learning.
(TIF)

Table S1 **Correlation coefficients between memory change scores and spindle differences (C4 minus C3).**
(TIF)

Table S2 **Correlation coefficients between spindle parameters and memory change scores.**
(XLSX)
