# Supplementary figures and images for: Susceptibility to Declarative Memory Interference is Pronounced in Primary Insomnia
Source: PLoS One. 2013 Feb 25;8(2):e57394. doi: 10.1371/journal.pone.0057394 (PMC3581453; doi:10.1371/journal.pone.0057394)

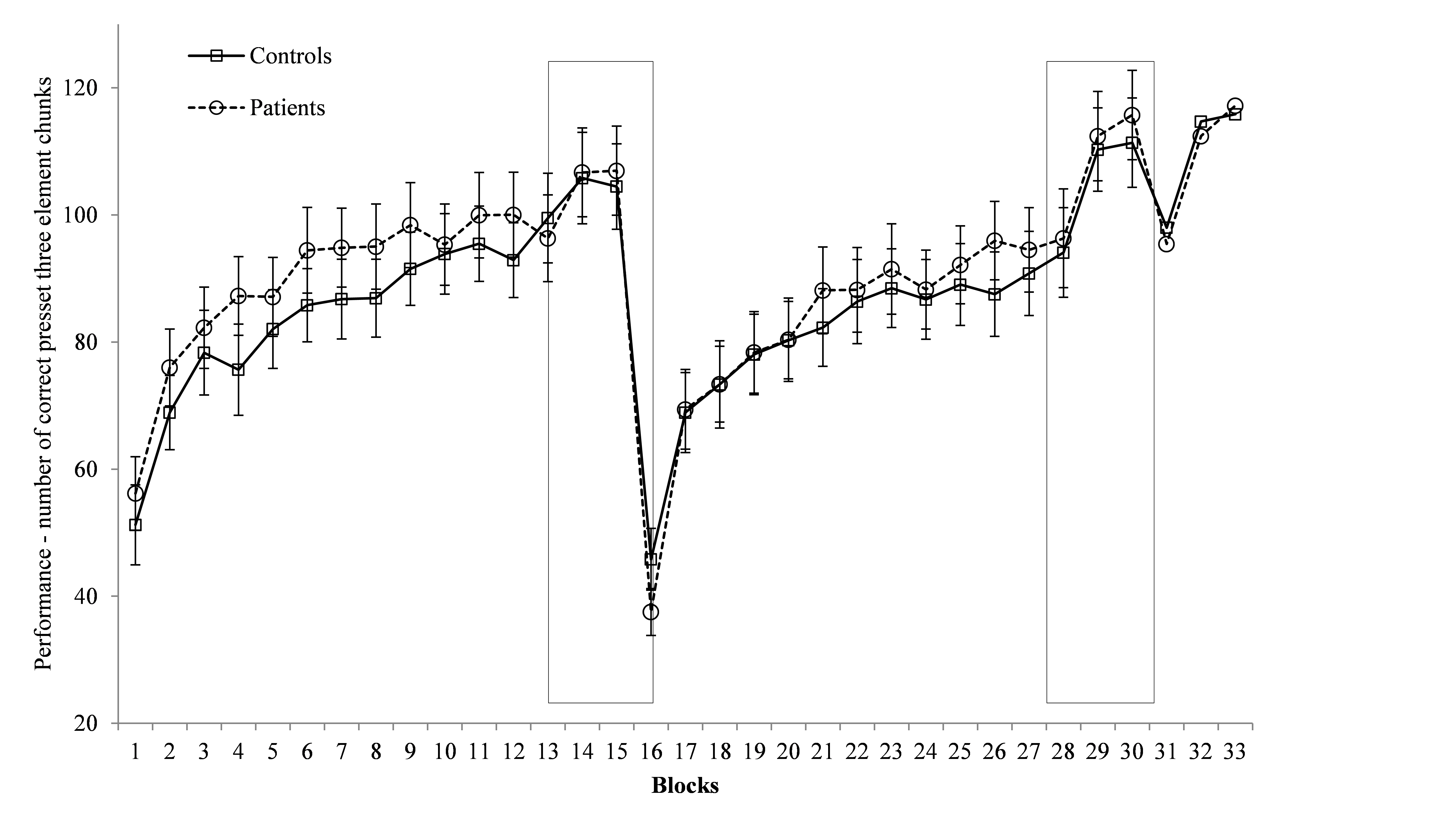

Supplement: Figure S1 — Performance during training (blocks 1–12), subsequent morning retest (blocks 13–15; in a box), interference testing (blocks 16–27), morning retest after interference (blocks 28–30; in a box) and follow up testing (blocks 31–33). Note that only a subgroup was tested in the follow up. (TIF) [file pone.0057394.s001.tif]

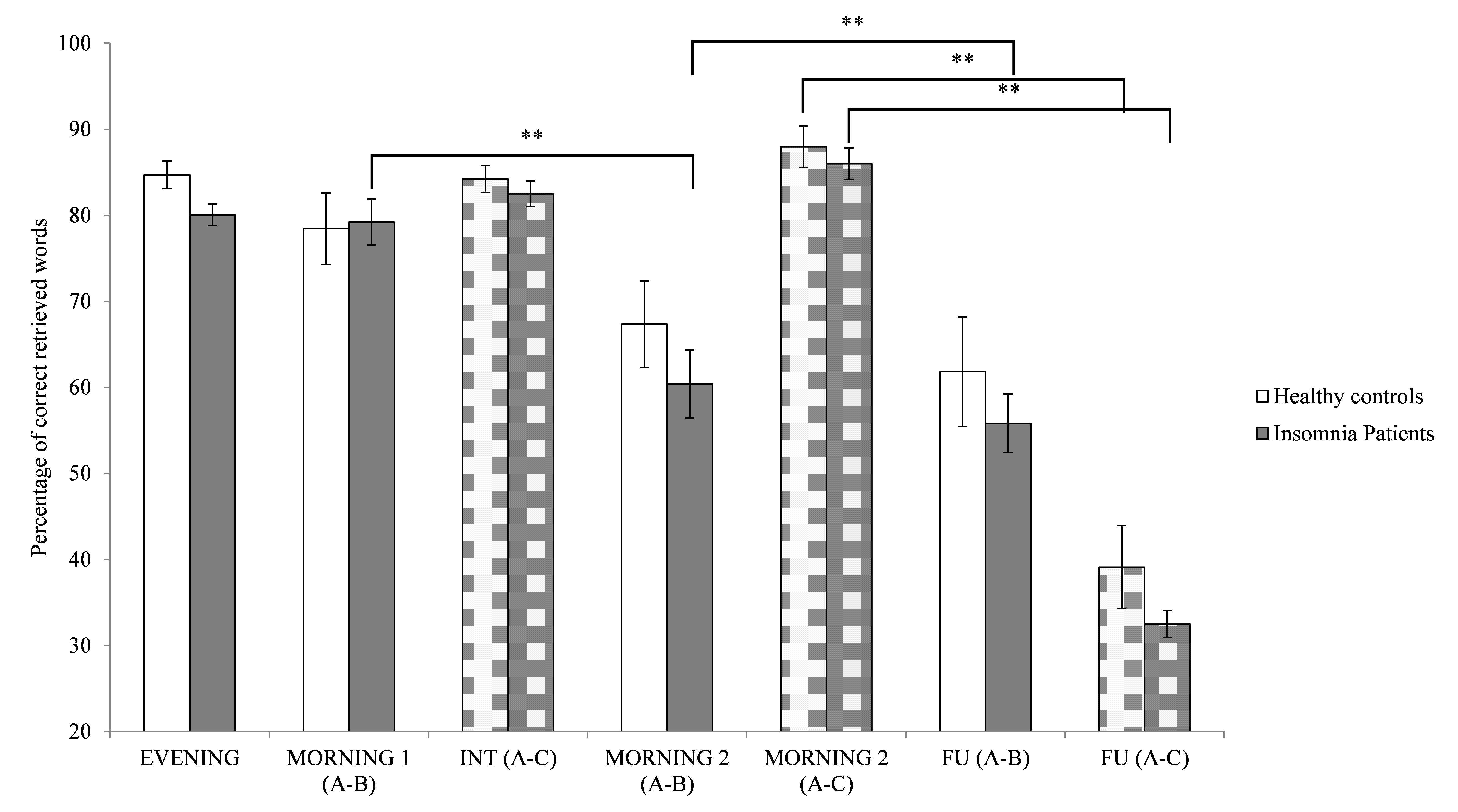

Supplement: Figure S2 — Declarative verbal memory scores from the evening, subsequent morning recall (MORNING 1), interference learning (INT), morning recall after interference (MORNING 2) and follow up (only a subgroup was tested) for all word lists (A–B, A–C). Bars represent means ± standard errors. Significant results are indicated by asterisks. **p<0.01. Note that only insomnia patients show significant forgetting after interference learning. (TIF) [file pone.0057394.s002.tif]

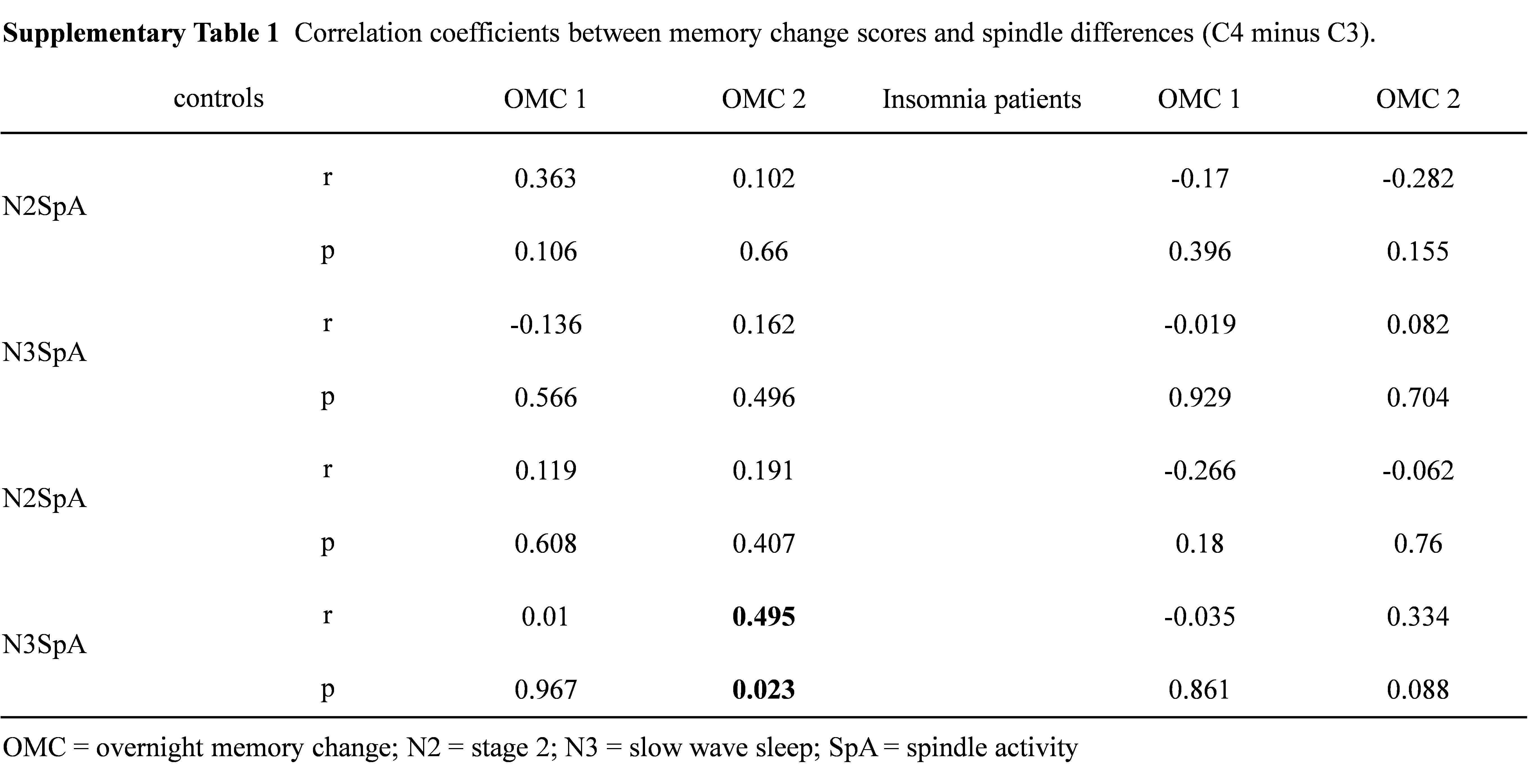

Supplement: Table S1 — Correlation coefficients between memory change scores and spindle differences (C4 minus C3). (TIF) [file pone.0057394.s003.tif]
